# Editorial: Special Issue “Edge and Fog Computing for Internet of Things Systems”

**DOI:** 10.3390/s22124387

**Published:** 2022-06-10

**Authors:** Behnam Dezfouli, Yuhong Liu

**Affiliations:** Department of Computer Science and Engineering, Santa Clara University, Santa Clara, CA 95053, USA; yhliu@scu.edu

Employing edge and fog computing for building IoT systems is essential, especially because of the massive number of data generated by sensing devices, the delay requirements of IoT applications, the high burden of data processing on cloud platforms, and the need to take immediate actions against security threats. By pushing processing and storage closer to IoT devices, it is possible to reduce the number of data sent to the cloud while also reducing communication delay. To this end, new data aggregation and processing methods are required to distribute computation across the edge-to-cloud continuum. Edge and fog computing can also be used to facilitate communication and resource discovery and enhance the security of IoT devices. New architectures are required to facilitate the communication between IoT devices and servers, depending on the type of application. From the data analytics point of view, efficient and scalable data processing at the edge or task offloading to trustworthy edge/fog nodes is critical to avoid significant delays and network congestion. Meanwhile, the massive and rapidly increasing number of resource-constrained IoT edge devices has significantly extended the attack surface, creating new challenges to ensuring data privacy and communication security against emerging threats and establishing trust among multiple communication parties.

This Special Issue has accepted nine papers and presents contributions to various aspects of the design, implementation, and evaluation of edge and fog computing architectures and systems in a wide variety of application domains, such as deep neural-network-based computation, ultra-dense networks, intelligent buildings, medical information systems, smart agriculture, and air quality monitoring. It showcases the challenging research issues and the promising future of the edge and fog computing era.

In [1], the authors propose a solution to address the computation offloading of DNN from mobile devices to edge clouds through a mobility-included DNN partition offloading algorithm (MDPO). The objective of the proposed algorithm is to minimize the total latency of the DNN computation when the mobile user is moving. In particular, the proposed algorithm can handle DNNs with both chain topology and DAG topology. There are two steps involved, where the first step converts the DAG topology to a chain topology and the second step constructs a DAG to represent the collaborative execution paths by the mobile device and the edge cloud. Then, the shortest path in the newly constructed DAG can be identified as the optimal partition offloading solution. 

Due to the resource constraints of edge clouds, edge servers are less reliable. The authors of [2] focus on reducing latency and improving the reliability of services in edge clouds. Specifically, they propose a fault-tolerant adaptive scheduling mechanism, which extends the traditional primary/backup (PB) model by introducing dynamic reliability requirements for tasks. They propose dynamically adjusting the start timing of task copies to improve resource utilization at the container level. Container resource is also adaptively adjusted based on the resource usage of assigned task copies.

The authors of [3] propose studying the load balancing issue among mobile edge computing (MEC) servers. In particular, the MEC scenario of ultra-dense networks (UDN) is considered. The authors propose adopting a software-defined network (SDN) to handle task allocation based on routing corresponding information between MEC servers. In addition, a load balancing algorithm based on user load prediction is proposed to resolve the NP-hard task offloading problem, which can effectively reduce the ping-pong effect. Furthermore, a genetic algorithm (GA) is implemented to evaluate the effectiveness and rapidity of the proposed algorithm. 

In [4], the authors focus on handling the application of big data processing with strict time requirements on fog computing nodes. Specifically, they propose a network-aware scheduling algorithm. Extending the Kubernetes default scheduler allows the network status to be taken into account, and it can identify the most suitable fog node for executing an application within a given deadline. 

In [5], the authors propose a queuing network-based message exchange architecture to evaluate the performance of intelligent building infrastructure equipped with IoT sensors and edge and fog devices, facilitating the design of computational architectures. A Design of Experiments (DoE) method is adopted to analyze the model’s sensitivity and identify bottlenecks. Three scenarios, including varying cores, varying number of fog nodes, and varying nodes and cores simultaneously, are simulated and studied. Several metrics are analyzed, including the average response time, resource utilization rate, flow rate, discard rate, and the number of messages in the system. 

In [6], the authors performed a systematic literature review of current studies in the smart agriculture domain on cloud, fog, and edge computing-based applications. In particular, the authors address several key questions: (1) How are new technologies such as cloud, fog, and edge used in smart agriculture, and what features of agriculture are covered? (2) What components are used in the architecture? (3) What type of combinations of computing is used? (4) What are the future direction and opportunities for smart agriculture using Cloud–Fog–Edge Computing? In the end, the authors also point out the remaining challenges and future directions.

In [7], the authors focus on medical information systems (MIS). The authors propose a comprehensive performability SRN model to quantify the performability of medical data transactions and services in local hospitals or medical centers. Specifically, the model adopts different failure modes and three main load-balancing techniques. Various performability metrics, including recover token rate, mean response time, drop probability, throughput, queue utilization of network devices, and fog nodes, are analyzed to evaluate the impact of load-balancing techniques and fail-over mechanisms. This study can facilitate improvements in the design of MIS systems. 

The authors of [8] propose a framework to offload the processing overhead of secure communication protocols from IoT devices to Wi-Fi access points (APs). The goal is to achieve maximum network throughput by utilizing the minimum number of APs while satisfying the security requirements within the APs’ computation and communication capacities. Specifically, this study presents a model for the device association problem as an optimization problem with a multi-objective function. The optimization problem is resolved via genetic algorithms (Gas) with constraints extracted from a physical testbed. 

In [9], the authors propose a methodology and an architectural framework for building large-scale sensing infrastructure for air quality monitoring in urban scenarios. The proposed solution is capable of handling the processing requirements of a large-scale application while sustaining real-time performance. The proposed scheme introduces methods to manage edge-tier node operation through different phases of the node life cycle, including the methods for node commission, provision, fault detection, and recovery. The sensor-side processing is encapsulated in the form of a microservices that reside on the different tiers of system architecture.

Finally, we would like to thank all the authors for their outstanding contributions and the reviewers for promptly providing their insightful review comments. We also sincerely appreciate the excellent support by the Editorial Board and Editorial Office of the MDPI Sensors journal.

## Data Availability

Not applicable.
